# The association of tooth loss, toothbrushing, and quality of life among cancer survivors

**DOI:** 10.1002/cam4.1835

**Published:** 2018-10-30

**Authors:** Rui Yan, Xuefen Chen, Xiaohuan Gong, Jiwei Wang, Jinming Yu

**Affiliations:** ^1^ Institute of Clinical Epidemiology, Key Laboratory of Public Health Safety, Ministry of Education, Key Lab of Health Technology Assessment of Ministry of Health, School of Public Health Fudan University Shanghai China; ^2^ Shanghai Municipal Center for Disease Control & Prevention Shanghai China

**Keywords:** cancer survivors, cross‐sectional studies, quality of life, tooth loss, toothbrushing

## Abstract

**Background:**

Tooth loss contributes physically and psychologically to health, and quality of life has been a key indicator of the cancer survivors. However, it is less clear whether tooth loss has impact on cancer survivors’ quality of life. Our study aimed to investigate the association between tooth loss, toothbrushing, and quality of life in cancer survivors.

**Methods:**

A cross‐sectional study was conducted among 9125 cancer survivors in Shanghai, China. Sociodemographic characteristics, frequency of tooth brushing, number of tooth loss were collected using a self‐reported questionnaire. Quality of life was measured using the EORTC QLQ‐C30. Chi‐square test was used to compare the distribution of tooth loss and toothbrushing frequency among various cancer sites, sociodemographic factors, socioeconomic status, health conditions. Multiple linear regression models were performed to estimate the effects of tooth loss and toothbrushing on quality of life.

**Results:**

Participants diagnosed with cancer of oral cavity, pharynx, and nasopharynx reported higher percentage of 11+ tooth loss. Cancer survivors with toothbrushing ≥2 times/d reported higher scores in physical, cognitive, and social function and had milder nausea and vomiting, compared with ones with toothbrushing <2 times/d. Tooth loss was associated with milder physical, role and emotional function scores, and severer fatigue, nausea/vomiting, dyspnea, insomnia, appetite loss, constipation, and diarrhea.

**Conclusions:**

This is the first study to investigate the impact of toothbrushing and tooth loss on quality of life among cancer survivors. Tooth loss was associated with milder physical, role and emotional function scores, and severer fatigue, nausea/vomiting, dyspnea, insomnia, appetite loss, constipation and diarrhea. Toothbrushing had significant positive effect on cancer survivors’ quality of life. The present study also provided several public health strategies to improve oral health among cancer survivors.

## 1 INTRODUCTION

It is estimated that a fifth of all global cancer living in China.^1^, ^2^ Cancer has become a serious public health problem threating people's health and it constitutes an enormous burden on the social development.^3^ Better primary health care, improved early detection, and effective treatment^4^ allowed individuals to live longer after cancer diagnosis,^2^ and cancer may be managed as a chronic illness.^4^ Despite these advance, cancer survivors still experienced great illness and psychological distress.^5^ Survival, as the primary end point, fails to sufficiently reflect the longer‐term physical and psychosocial effects for cancer survivors. More attention has been paid to evaluate the cancer survivors’ quality of life (QOL),^6^ a multidimensional concept covering various aspects, including physical, emotional, mental, sexual, and social functioning.^7^ QOL reflects the individual's experience about the survival‐related goals^8^, ^9^ and had been recognized as an important prognostic variable and widely used in cancer research.^7^

Oral cavity acts as a window into individuals’ body health and shows signs of nutritional deficiencies or general infection. Oral health is an important part of individuals’ overall health, and good oral health should include the absence of facial pain, proper chewing, and convenient ingesting.^10^ Oral health affects gastrointestinal flora and nutritional status.^11^ Cancer therapies may cause acute and late oral complications on cancer survivors,^12^ including mucositis, infection, saliva and neurosensory changes, and taste alteration,^13^ which may affect health‐related QOL.^14^

Tooth loss is one of the common oral health measures. Tooth loss is associated with various factors, including age, smoking, drinking, dental diseases, poverty, faulty nutrition, and much more.^10^ Cancer patients undergoing radiotherapy^15^, ^16^ and chemotherapy^17^ may experience some unwanted oral side effects, and result in higher risk of tooth loss. Individuals with missing teeth lose some orofacial structures, such as bone tissues, nerves, receptors and muscles, and in decreased orofacial functions.^18^Many epidemiologic studies also indicated the potential association of tooth loss with higher cancer risk.^19^, ^20^, ^21^ Poor oral hygiene and the following tooth loss might result in greater carcinogens production, specifically nitrosamines, and increased risk of cancers.^11^ Tooth loss influences the food choice, nutrition intake, and dissatisfaction with appearance and also has the potential to impair individuals’ QOL. Some previous studies found that tooth loss was associated with health‐related quality of life (HRQOL)^22^ and oral health‐related quality of life OHRQOL among adults.^23^ However, no research was found to investigate the impact of tooth loss on cancer survival patients’ overall QOL, which including physical, emotional, mental, sexual, and social functioning.

Toothbrushing is a daily means to maintain oral health and is closely related to oral health and hygiene.^24^ Since the good oral health was associated with decreased cancer risk and improved QOL, it might be supposed that toothbrushing has the similar relationship. It has been published in several researches that toothbrushing frequency was associated with head and neck,^25^ esophageal^26^ and upper aerodigestive tract cancer.^24^ However, there are limited data on toothbrushing frequency and QOL among cancer survivors.

Therefore, the present study described the current tooth loss and toothbrushing frequency status in Chinese cancer survivors and evaluated their association with QOL using EORTC QLQ‐C30, a cancer‐special multiple dimension scale of QOL. Insight in this association will serve to identify goals for oral health to improve cancer survivors’ QOL.

## 2 MATERIALS AND METHODS

### 2.1 Participants

This cross‐sectional study was conducted in Shanghai Cancer Rehabilitation Club and recruited cancer survivors from community and hospitals covering all 17 counties of Shanghai. Inclusion criteria included pathological diagnosis, able to independently participate in the activities of cancer rehabilitation club, and without cognitive impairment. Data was collected using a self‐reported structured questionnaire including questions about basic sociodemographic factors (age, gender, and marital status), socioeconomic status (education level, occupation, and income), life behavior (smoking and drinking), health conditions (BMI, comorbidity, treatment, and time since diagnosis), oral health (frequency of toothbrushing, and number of tooth loss), and QOL.

In total, 9569 adults were invited to participate in our study. Field workers checked questionnaires in time, and 444 questionnaires were determined as incomplete due to the large proportion of missing data. At last, a total of 9125 (95.36%) cancer questionnaires were included in the current study. Informed consent was obtained from each study participant. The study was approved by the Medical Research Ethics Committee of the school of public health, Fudan University (The international registry NO. IRB00002408 & FWA00002399).

### 2.2 QOL measurement

Quality of life was evaluated by the European Organization for Research and Treatment of Cancer Quality of Life Questionnaire‐Core30 (EORTC QLQ‐C30) simplified Chinese V3.0 version, which had been widely used in the study of Chinese cancer patients with acceptable reliability, validity, and sensitivity.^27^ It reflects multiple dimensions of QOL, including functional scales (physical, role, cognitive, emotional, social), symptom scales (fatigue, nausea and vomiting, pain), global HRQL scale and six single items (dyspnea, insomnia, appetite loss, constipation, diarrhea, and financial difficulties).^28^ According to the EORTC QLQ‐C30 Scoring Manual,^28^ crude subscale scores are transformed to standard scores ranged from 0 to 100. For functional and global health scales, a higher score represents a better level of functioning. For symptom and financial scales, higher scores represent more severe symptoms.

### 2.3 Oral health

The frequency of toothbrushing and the number of tooth loss were collected in the self‐reported questionnaire. The number of teeth loss was recorded as absent teeth (ie, missing due to caries, extracted, congenitally absent, or unerupted). Number of tooth loss was determined by the question ‘‘How many of your missing teeth do you have?’’ The number of tooth loss was categorized into four groups (0, 1‐5, 6‐10, and ≥11). Brushing status was determined by the question ‘‘How often do you usually brush your teeth?’’ Participants could choose from the following: ≥2 times/d, 1 time/d and without brush your teeth per day.

### 2.4 Statistical analysis

Means and standard deviations were calculated for continuous variables, and numbers and percentages were computed for categorical variables. The chi‐square test was used to compare the differences in distribution of tooth loss among the ten kinds of main cancer sites, with adjusted α value (α/45).The distribution of tooth loss and the frequency of toothbrushing among different sociodemographic factors, socioeconomic status, and health conditions were compared using chi‐square test. Multiple linear regression models were performed to estimate the mean differences and 95% CI of QOL scores, adjusted for age, BMI, education, marital status, household per capita income, smoking, drinking, time since diagnosis, treatment, and comorbidities. Trend test was performed by entering the tooth loss groups as continuous data in models. All statistical analyses were performed by Statistical Analysis Software (SAS) version 9.4 (SAS Institute, Cary, NC, USA). A two‐sided *P* value <0.05 or 0.05/45 (for the paired‐comparisons of chi‐square test) was considered as the significant level.

## 3 RESULTS

### 3.1 Cancer site and tooth loss

In our study, 3396 (37.22%) cancer patients were diagnosed with breast cancer, and the second most common diagnose was digestive system neoplasm (2725, 29.86%). The other 286 (3.13%) patients were diagnosed with cancer of oral cavity, pharynx, and nasopharynx, and among them, the percentage of the reports of 11+ tooth loss was 33.57%, which was significantly higher than those with cancers of digestive organ (17.69%), respiratory organs and thorax (18.95%), breast (10.25%), female genital organs (12.05%), urinary tract (16.62%), and thyroid (11.83%). Patients with cancer of male genital organs were older (71.48 ± 9.53 years) those with other cancers, and reported higher percentage of 11 + tooth loss (29.47%) than those with cancer of respiratory organs and thorax (18.95%), breast (10.25%), female genital organs (12.05%), and thyroid (11.83%; Table 1).

**Table 1:** Number of tooth loss and cancer sites

Cancer site	N (%)	Age	Number of tooth loss			
			0	1‐5	6‐10	≥11
Oral cavity, Pharynx and Nasopharynx	286 (3.13%)	61.20 ± 9.13	25 (8.74%)	92 (32.17%)	73 (25.52%)	96 (33.57%)
Oral cavity and pharynx	71 (0.78%)	65.62 ± 8.52	3 (4.23%)	17 (23.94%)	19 (26.76%)	32 (45.07%)
Nasopharynx	215 (2.36%)	59.74 ± 8.87	22 (10.23%)	75 (34.88%)	54 (25.12%)	64 (29.77%)
Digestive organ^a^	2725 (29.86%)	63.86 ± 8.77	281 (10.31%)	1123 (41.21%)	839 (30.79%)	482 (17.69%)
Esophagus	66 (0.72%)	65.11 ± 9.97	3 (4.55%)	23 (34.85%)	21 (31.82%)	19 (28.79%)
Stomach	979 (10.73%)	63.39 ± 9.03	101 (10.32%)	401 (40.96%)	291 (29.72%)	186 (19%)
Colon‐rectum	1407 (15.42%)	64.48 ± 8.67	154 (10.95%)	578 (41.08%)	439 (31.2%)	236 (16.77%)
Liver	222 (2.43%)	62.15 ± 7.33	19 (8.56%)	103 (46.4%)	68 (30.63%)	32 (14.41%)
Gallbladder	20 (0.22%)	65.05 ± 6.3	3 (15%)	6 (30%)	6 (30%)	5 (25%)
Pancreas	31 (0.34%)	59.10 ± 9.79	1 (3.23%)	12 (38.71%)	14 (45.16%)	4 (12.9%)
Respiratory organs and thorax^a^^,^ b	839 (9.19%)	63.44 ± 8.39	83 (9.89%)	328 (39.09%)	269 (32.06%)	159 (18.95%)
Larynx	118 (1.29%)	65.93 ± 9.41	4 (3.39%)	31 (26.27%)	42 (35.59%)	41 (34.75%)
Lung	707 (7.75%)	63.09 ± 8.10	78 (11.03%)	291 (41.16%)	222 (31.4%)	116 (16.41%)
Other thoracic organs	14 (0.15%)	60.29 ± 10.58	1	6	5	2
Breast^a^^,^ b ^,^ c	3396 (37.22%)	59.25 ± 7.99	476 (14.02%)	1654 (48.7%)	918 (27.03%)	348 (10.25%)
Female genital organs^a^^,^ b ^,^ c ^,^ d	639 (7%)	58.39 ± 9.02	75 (11.74%)	312 (48.83%)	175 (27.39%)	77 (12.05%)
Cervix	224 (2.45%)	56.00 ± 10.04	30 (13.39%)	107 (47.77%)	59 (26.34%)	28 (12.5%)
Uterus	102 (1.12%)	60.03 ± 8.74	9 (8.82%)	57 (55.88%)	27 (26.47%)	9 (8.82%)
Ovary	300 (3.29%)	59.33 ± 7.92	34 (11.33%)	144 (48%)	86 (28.67%)	36 (12%)
Male genital organs^d^^,^ e ^,^ f	95 (1.04%)	71.48 ± 9.53	7 (7.37%)	34 (35.79%)	26 (27.37%)	28 (29.47%)
Prostate	88 (0.96%)	72.45 ± 7.60	5 (5.68%)	33 (37.5%)	24 (27.27%)	26 (29.55%)
Testis	7 (0.08%)	59.29 ± 19.92	2	1	2	2
Urinary tract^a^^,^ e	337 (3.69%)	64.36 ± 8.86	31 (9.2%)	145 (43.03%)	105 (31.16%)	56 (16.62%)
Kidney	210 (2.3%)	63.08 ± 7.56	23 (10.95%)	101 (48.1%)	56 (26.67%)	30 (14.29%)
Bladder	127 (1.39%)	66.49 ± 10.37	8 (6.3%)	44 (34.65%)	49 (38.58%)	26 (20.47%)
Thyroid^a^^,^ b ^,^ c ^,^ d	279 (3.06%)	57.90 ± 8.52	43 (15.41%)	137 (49.1%)	66 (23.66%)	33 (11.83%)
Lymphatic and hematopoietic system^e^	234 (2.56%)	58.91 ± 9.57	30 (12.82%)	97 (41.45%)	59 (25.21%)	48 (20.51%)
Lymphoma	164 (1.8%)	59.59 ± 9.84	22 (13.41%)	60 (36.59%)	43 (26.22%)	39 (23.78%)
Leukemia	70 (0.77%)	57.33 ± 8.78	8 (11.43%)	37 (52.86%)	16 (22.86%)	9 (12.86%)
All others^a^^,^ g	295 (3.23%)	60.73 ± 9.28	22 (7.46%)	125 (42.37%)	96 (32.54%)	52 (17.63%)
Brain	35 (0.38%)	54.51 ± 10.29	4 (11.43%)	17 (48.57%)	12 (34.29%)	2 (5.71%)
Bone	14 (0.15%)	62.07 ± 7.02	2	3	7	2
Melanoma of Skin	10 (0.11%)	64.10 ± 11.27	0	7	1	2
Other cancers	249 (2.73%)	61.61 ± 8.78	18 (7.23%)	102 (40.96%)	79 (31.73%)	50 (20.08%)
All sites	9125 (100%)	61.32 ± 8.90	1073 (11.76%)	4047 (44.35%)	2626 (28.78%)	1379 (15.11%)

The differences in the distributions of the number of tooth loss among various cancer sites were evaluated by chi‐square test, with adjusted *P* value.

a Different with oral cavity, pharynx and nasopharynx.

b Different with male genital organs.

c Different with digestive organ.

d Different with respiratory organs and thorax.

e Different with breast.

f Different with female genital organs.

g Different with thyroid.

### 3.2 Basic characteristics, toothbrushing, and tooth loss

Among the 9125 cancer survivors (2725 male, 6400 female), 57.05% of them were aged 60 years or older. Most participants were married (88.42%), and 4875 (53.42%) participants had attained a high school or higher education. 77.05% participants had one or more comorbidities, and 61.62% participants had survival more than 5 years since diagnosis. Brushing tooth at least 2 times/d was reported by 55.63% participants, 15.11% cancer survivors reported more than 10 missing teeth. Only 641 (7.02%) cancer survivors reported dental visit more than 1 time/y (Table 2).

**Table 2:** Number of tooth loss, frequency of toothbrushing, and basic characteristics

Characteristics	Total（N = 9125）	The number of tooth loss					Toothbrushing		
		0（N = 1073）	1‐5（N = 4047）	6‐10（N = 2626）	≥11（N = 1379）	*P*	≤1 times/d（N = 4058）	≥2 times/d（N = 5076）	*P*
Gender									
Male	2725 (29.86%)	250 (9.17%)	1027 (37.69%)	870 (31.93%)	578 (21.21%)		1427 (52.37%)	1298 (47.63%)	
Female	6400 (70.14%)	823 (12.86%)	3020 (47.19%)	1756 (27.44%)	801 (12.52%)	**<0.001**	2631 (41.11%)	3769 (58.89%)	**<0.001**
Age (y)									
<50	605 (6.63%)	176 (29.09%)	285 (47.11%)	122 (20.17%)	22 (3.64%)		270 (44.63%)	335 (55.37%)	
50~	3314 (36.32%)	500 (15.09%)	1704 (51.42%)	858 (25.89%)	252 (7.6%)		1461 (44.09%)	1853 (55.91%)	
≥60	5206 (57.05%)	397 (7.63%)	2058 (39.53%)	1646 (31.62%)	1105 (21.23%)	**<0.001**	2327 (44.7%)	2879 (55.3%)	0.855
BMI (kg/m^2^)									
<18.5	482 (5.28%)	47 (9.75%)	170 (35.27%)	153 (31.74%)	112 (23.24%)		199 (41.29%)	283 (58.71%)	
18.5~	6228 (68.25%)	766 (12.3%)	2782 (44.67%)	1776 (28.52%)	904 (14.52%)		2632 (42.26%)	3596 (57.74%)	
25.0~	2198 (24.09%)	242 (11.01%)	999 (45.45%)	623 (28.34%)	334 (15.2%)		1107 (50.36%)	1091 (49.64%)	
≥30.0	217 (2.38%)	18 (8.29%)	96 (44.24%)	74 (34.1%)	29 (13.36%)	**<0.001**	120 (55.3%)	97 (44.7%)	**<0.001**
Education									
<High school	4250 (46.58%)	462 (10.87%)	1847 (43.46%)	1272 (29.93%)	669 (15.74%)		2277 (53.58%)	1973 (46.42%)	
High school	3382 (37.06%)	428 (12.66%)	1540 (45.54%)	965 (28.53%)	449 (13.28%)		1330 (39.33%)	2052 (60.67%)	
>High school	1493 (16.36%)	183 (12.26%)	660 (44.21%)	389 (26.05%)	261 (17.48%)	**<0.001**	451 (30.21%)	1042 (69.79%)	**<0.001**
Marital status									
Married	8068 (88.42%)	967 (11.99%)	3649 (45.23%)	2304 (28.56%)	1148 (14.23%)		3583 (44.41%)	4485 (55.59%)	
Unmarried/widowed/divorced	1057 (11.58%)	106 (10.03%)	398 (37.65%)	322 (30.46%)	231 (21.85%)	**<0.001**	475 (44.94%)	582 (55.06%)	0.745
Number of comorbidity									
0	2094 (22.95%)	350 (16.71%)	859 (41.02%)	644 (30.75%)	241 (11.51%)		993 (47.42%)	1101 (52.58%)	
1	2148 (23.54%)	269 (12.52%)	1003 (46.69%)	576 (26.82%)	300 (13.97%)		955 (44.46%)	1193 (55.54%)	
2	1989 (21.8%)	217 (10.91%)	899 (45.2%)	597 (30.02%)	276 (13.88%)		872 (43.84%)	1117 (56.16%)	
≥3	2894 (31.72%)	237 (8.19%)	1286 (44.44%)	809 (27.95%)	562 (19.42%)	**<0.001**	1238 (42.78%)	1656 (57.22%)	**0.011**
Household per capita income (yuan/y)									
<2000	2273 (24.91%)	263 (11.57%)	990 (43.55%)	664 (29.21%)	356 (15.66%)		1337 (58.82%)	936 (41.18%)	
2000~	5263 (57.68%)	600 (11.4%)	2356 (44.77%)	1532 (29.11%)	775 (14.73%)		2144 (40.74%)	3119 (59.26%)	
≥4000	1589 (17.41%)	210 (13.22%)	701 (44.12%)	430 (27.06%)	248 (15.61%)	0.297	577 (36.31%)	1012 (63.69%)	**<0.001**
Drinking									
No	7939 (87%)	982 (12.37%)	3506 (44.16%)	2311 (29.11%)	1140 (14.36%)		3453 (43.49%)	4486 (56.51%)	
Yes	1186 (13%)	91 (7.67%)	541 (45.62%)	315 (26.56%)	239 (20.15%)	**<0.001**	605 (51.01%)	581 (48.99%)	**<0.001**
Smoking									
No	8677 (95.09%)	1030 (11.87%)	3870 (44.6%)	2498 (28.79%)	1279 (14.74%)		3789 (43.67%)	4888 (56.33%)	
Yes	448 (4.91%)	43 (9.6%)	177 (39.51%)	128 (28.57%)	100 (22.32%)	**<0.001**	269 (60.04%)	179 (39.96%)	**<0.001**
Time since diagnosis (y)									
<2	1053 (11.54%)	155 (14.72%)	472 (44.82%)	301 (28.58%)	125 (11.87%)		512 (48.62%)	541 (51.38%)	
2~	2449 (26.84%)	333 (13.6%)	1186 (48.43%)	663 (27.07%)	267 (10.9%)		1131 (46.18%)	1318 (53.82%)	
5~	2774 (30.4%)	309 (11.14%)	1251 (45.1%)	808 (29.13%)	406 (14.64%)		1270 (45.78%)	1504 (54.22%)	
≥10	2849 (31.22%)	276 (9.69%)	1138 (39.94%)	854 (29.98%)	581 (20.39%)	**<0.001**	1145 (40.19%)	1704 (59.81%)	**<0.001**
Surgery									
No	1203 (13.18%)	135 (11.22%)	495 (41.15%)	352 (29.26%)	221 (18.37%)		571 (47.46%)	632 (52.54%)	
Yes	7922 (86.82%)	938 (11.84%)	3552 (44.84%)	2274 (28.7%)	1158 (14.62%)	**0.003**	3487 (44.02%)	4435 (55.98%)	**0.025**
Radiotherapy									
No	6310 (69.15%)	745 (11.81%)	2768 (43.87%)	1827 (28.95%)	970 (15.37%)		2866 (45.42%)	3444 (54.58%)	
Yes	2815 (30.85%)	328 (11.65%)	1279 (45.44%)	799 (28.38%)	409 (14.53%)	0.521	1192 (42.34%)	1623 (57.66%)	**0.006**
Chemotherapy									
No	2361 (25.87%)	259 (10.97%)	962 (40.75%)	715 (30.28%)	425 (18%)		1112 (47.1%)	1249 (52.9%)	
Yes	6764 (74.13%)	814 (12.03%)	3085 (45.61%)	1911 (28.25%)	954 (14.1%)	**<0.001**	2946 (43.55%)	3818 (56.45%)	**0.003**
TCM									
No	4848 (53.13%)	537 (11.08%)	2092 (43.15%)	1467 (30.26%)	752 (15.51%)		2298 (47.4%)	2550 (52.6%)	
Yes	4277 (46.87%)	536 (12.53%)	1955 (45.71%)	1159 (27.1%)	627 (14.66%)	**<0.001**	1760 (41.15%)	2517 (58.85%)	**<0.001**
Toothbrushing									
≤1 time/d	4058 (44.15%)	463 (11.41%)	1730 (42.63%)	1182 (29.13%)	683 (16.83%)				
≥2 times/d	5076 (55.63%)	610 (12.04%)	2317 (45.73%)	1444 (28.5%)	696 (13.74%)	**<0.001**			
Dental visit									
<1 time/y	8484 (92.98%)	995 (11.73%)	3784 (44.60%)	2448 (28.85%)	1257 (14.82%)				
≥1 time/y	641 (7.02%)	78 (12.17%)	263 (41.03%)	178 (27.77%)	122 (19.03%)	**0.028**			

The differences in the distributions of the number of tooth loss and the frequency of toothbrushing among various basic characteristics were evaluated by chi‐square test. Bold face *P* < 0.05

TMC, traditional Chinese medicine.

Female cancer survivors reported lower percentage of 6 + tooth loss (39.96%) and higher percentage of toothbrushing ≥2 times/d (58.89%) than male. Older cancer survivors reported higher percentage of 6 + tooth loss. Cancer survivors with higher levels of education (>high school) or income (>2000 yuan/mo) reported higher percentage of toothbrushing ≥2 times/d. Cancer survivors with more than three comorbidities reported the highest percentage of 11+ tooth loss (19.42%). Smoker and drinker reported lower percentage of 11+ tooth loss and lower percentage of toothbrushing ≥2 times/d than nonsmoker and nondrinker. The distribution of tooth loss was different between cancer survivors with the treatment of surgery, chemotherapy, or traditional Chinese medicine and those without these treatments. More frequent toothbrushing (≥2 times/d) reported lower percentage of 6+ tooth loss than those toothbrushing ≤1 time/d (Table 2).

### 3.3 Toothbrushing and QOL

The influence of toothbrushing frequency on EORTC QLQ‐C30 scores was presented in Table 3. Participants with ≥2 times/d toothbrushing reported significant higher physical score (MD = 0.88, 95% CI: 0.21‐1.54, *P* = 0.01), cognitive score (MD = 1.08, 95% CI: 0.29‐1.87, *P* = 0.008), social function score (MD = 1.21, 95% CI: 0.23‐2.20, *P* = 0.016), lower nausea and vomiting score (MD = −0.66, 95% CI: −1.16 to −0.17, *P* = 0.009) and financial difficulties score (MD = −1.86, 95% CI: −3.13 to −0.59, *P* < 0.001) than those with toothbrushing ≤1 time/d.

**Table 3:** Associations between frequency of toothbrushing and quality of life in cancer survivors

Scales	Average crude score [Mean (SD)]		Adjusted mean difference of scores (95% CI)^a^	*P* a
	Toothbrushing ≤1 time/d (N = 4058)	Toothbrushing ≥2 times/d (N = 5067)		
EORTC QLQ‐C30				
Physical function	81.32 ± 16.36	81.34 ± 15.55	**0.88 (0.21, 1.54)**	**0.010**
Role function	89.39 ± 18.73	88.99 ± 18.96	0.13 (−0.68, 0.93)	0.755
Emotional function	84.51 ± 17.15	84.54 ± 17.39	0.28 (−0.45, 1.00)	0.457
Cognitive function	77.85 ± 19.20	79.04 ± 18.54	**1.08 (0.29, 1.87)**	**0.008**
Social function	76.36 ± 23.84	77.93 ± 23.00	**1.21 (0.23, 2.20)**	**0.016**
Global health/QoL	61.94 ± 25.22	61.94 ± 22.96	0.97 (−0.14, 2.08)	0.088
Fatigue	28.88 ± 20.35	29.61 ± 19.57	−0.37 (−1.20, 0.45)	0.376
Nausea and vomiting	4.28 ± 12.37	3.59 ± 10.80	−**0.66 (−1.16,** −**0.17)**	**0.009**
Pain	17.53 ± 19.79	18.23 ± 19.75	0.34 (−0.48, 1.16)	0.417
Dyspnoea	16.23 ± 20.92	16.36 ± 20.37	−0.16 (−1.04, 0.72)	0.719
Insomnia	19.28 ± 24.32	20.92 ± 24.44	0.44 (−0.59, 1.47)	0.404
Appetite loss	10.48 ± 19.16	9.80 ± 18.07	−0.67 (−1.46, 0.12)	0.098
Constipation	11.57 ± 20.15	11.88 ± 20.53	−0.15 (−1.02, 0.72)	0.729
Diarrhea	8.60 ± 17.26	8.40 ± 16.89	−0.35 (−1.08, 0.39)	0.357
Financial difficulties	33.77 ± 31.64	29.82 ± 30.07	−**1.86 (−3.13,** −**0.59)**	**<0.001**

Bold face *P* < 0.05

a Adjusted for age, BMI, education, marital status, household per capita income, smoking, drinking, time since diagnosis, treatment, comorbidities.

### 3.4 Tooth loss and QOL

Table 4 presented the influence of tooth loss on EORTC QLQ‐C30 scores. Compared with the participants without missing teeth, the participants with missing tooth reported significantly milder physical function, role function and emotional function scores, and higher dyspnoea score. With the increase of the number of missing tooth, participants reported higher scores of fatigue (*P*_trend _= 0.019), nausea/vomiting (*P*_trend _< 0.001), dyspnea (*P*_trend _< 0.001), insomnia (*P*_trend _= 0.03), appetite loss (*P*_trend _< 0.001), constipation (*P*_trend _< 0.001), and diarrhea (*P*_trend _= 0.016).

**Table 4:** Associations between number of tooth loss and quality of life in cancer survivors

Scales	Average crude score [Mean (SD)]	Adjusted mean difference of scores (95% CI) vs none						
	None (N = 1073)	1‐5 (N = 4047)	*P* a	6‐10 (N = 2626)	*P* a	≥11 (N = 1379)	*P* a	*P*_trend_
EORTC QLQ‐C30								
Physical function	83.80 ± 14.72	**−1.15 (−2.19, −0.11)**	**0.030**	**−1.70 (−2.82, −0.58)**	**0.003**	−**2.74 (−4.04, −1.44)**	**<0.001**	**<0.001**
Role function	91.25 ± 16.69	−**1.39 (−2.66, −0.13)**	**0.031**	−**1.51 (−2.87, −0.16)**	**0.029**	−**1.86 (−3.44, −0.29)**	**0.020**	**0.045**
Emotional function	85.89 ± 16.58	−**1.32 (−2.47, −0.18)**	**0.023**	−**1.82 (−3.05, −0.6)**	**0.004**	−**2.5 (−3.92, −1.07)**	**0.001**	**<0.001**
Cognitive function	80.89 ± 17.85	−**1.39 (−2.63, −0.14)**	**0.029**	−0.84 (−2.17, 0.50)	0.219	−**2.39 (−3.94, −0.84)**	**0.003**	**0.036**
Social function	77.05 ± 23.96	0.16 (−1.40, 1.71)	0.844	0.55 (−1.11, 2.21)	0.517	−1.26 (−3.2, 0.67)	0.200	0.316
Global health/QoL	64.86 ± 22.81	−**1.76 (−3.50, −0.02)**	**0.047**	−**2.13 (−4.02, −0.23)**	**0.028**	−1.34 (−3.52, 0.84)	0.229	0.298
Fatigue	27.36 ± 19.04	**1.61 (0.31, 2.91)**	**0.015**	0.82 (−0.57, 2.21)	0.249	**2.89 (1.27, 4.51)**	**0.001**	**0.019**
Nausea and vomiting	3.25 ± 10.30	0.30 (−0.47, 1.08)	0.444	**0.95 (0.12, 1.78)**	**0.025**	**1.31 (0.34, 2.28)**	**0.008**	**<0.001**
Pain	15.51 ± 18.69	**1.42 (0.13, 2.71)**	**0.032**	1.24 (−0.15, 2.62)	0.080	1.31 (−0.30, 2.92)	0.111	0.300
Dyspnoea	13.49 ± 19.27	**1.52 (0.14, 2.90)**	**0.031**	**1.87 (0.39, 3.35)**	**0.013**	**2.99 (1.27, 4.71)**	**0.001**	**<0.001**
Insomnia	17.67 ± 23.24	**2.21 (0.59, 3.83)**	**0.007**	1.39 (−0.34, 3.13)	0.116	**3.35 (1.34, 5.37)**	**0.001**	**0.030**
Appetite loss	8.36 ± 17.29	0.75 (−0.49, 2.00)	0.235	**1.42 (0.08, 2.75)**	**0.037**	**2.79 (1.24, 4.34)**	**0.000**	**<0.001**
Constipation	9.87 ± 19.41	0.65 (−0.72, 2.02)	0.354	**1.69 (0.22, 3.16)**	**0.024**	**1.91 (0.21, 3.62)**	**0.028**	**<0.001**
Diarrhea	7.34 ± 15.88	0.57 (−0.59, 1.73)	0.336	0.82 (−0.42, 2.06)	0.195	**1.76 (0.32, 3.20)**	**0.017**	**0.016**
Financial difficulties	33.65 ± 32.64	−0.40 (−2.40, 1.60)	0.694	−1.59 (−3.74, 0.55)	0.145	0.27 (−2.21, 2.76)	0.829	0.709

Bold face *P* < 0.05

a Adjusted for age, BMI, education, marital status, household per capita income, time since diagnosis, smoking, drinking, treatment, comorbidities, frequency of toothbrushing.

## 4 DISCUSSION

The present study provided evidence that age, smoking, drinking and comorbidities were significantly associated with higher percentage of tooth loss in cancer survivors. Frequent toothbrushing could have a protective effect for tooth loss. In addition, cancer survivors with severe tooth loss and poor oral hygiene habit might experience worse QOL.

Tooth loss is an effective marker of oral health and increases gradually with age.^29^ Our results were consistent with the conclusions of previous researches that smoking, drinking, and level of education were important determinants of tooth loss.^30^, ^31^, ^32^ Cancer survivors diagnosed with cancers of oral cavity, pharynx, and nasopharynx reported more tooth loss than other types of cancers. The dental care should play an important and proactive role in cancer survival care,^15^ especially for oral cavity, pharynx, and nasopharynx cancer patients.

Our results indicated that better oral health, such as toothbrushing ≥2 times/d, was associated with lower percentage of tooth loss, had a protective effect for nausea and vomiting, and was beneficial for physical, cognitive, and social function. Toothbrushing is a daily means to maintain oral health, it can clean out the food debris, reduce microorganisms and inflammation.^24^ The less frequent of toothbrushing may contribute to the increased prevalence of periodontal disease and dental caries. Dental caries is the primary cause of tooth loss,^33^ and severe periodontal disease could result in tooth loosening and eventual tooth loss.^34^ Teeth brushing helps freshen individuals’ breath, avoid the embarrassment in communication and working and is beneficial for QOL for cancer survivors. Nausea and vomiting are the common clinical manifestation of anti‐tumor therapy for cancer patients, and have negative influence on therapy compliance. Frequent toothbrushing could decrease the nausea and vomiting and provide guarantees for the successful tumor treatment.

The present study provided an important look at tooth loss and QOL in cancer survivors, and tooth loss was found to be associated with poorer function scores and higher symptom scores. Tooth loss could impact on general health physiologically and psychologically. Tooth loss was associated with reduced masticatory function, chewing ability, food selection, diet and nutrition.^35^ Subjects with an incomplete dentition may choose to swallow food rather than chew more times,^35^ which may result in malnutrition, reduced immune function and affect the anticancer treatment.^36^ Tooth loss could also impair individual's self‐esteem, self‐image, self‐satisfaction and well‐being,^37^ and have influence on QOL.^38^ Since cancer survivors endure a long‐term of illness, and suffer various degrees of psychological stress, the additional problem of tooth loss may increase their psychological burden and physical discomfort. The preventive measures to tooth loss for cancer patients are important and should be highlighted, not only as a way to improve oral health but also as a tool to increase QOL benefit.

Tooth loss may be a complication of some anticancer therapy, and the dental care should play an important, proactive role in the overall cancer survival care.^15^ In our study, only 641 (7.02%) cancer survivors reported dental visit more than 1 time/year, far less than Australia (63.7%) in a 2‐year prospective cohort study.^39^ Cancer survivorship is associated with substantial medical expenditures.^40^ Oral health care is costly; however, in China, the basic medical insurance only covers a small part of dental health care expenditures,^41^ and patients should pay more than 85% of the total dental expense^42^ and the treatment of prosthodontics is not cover by insurance.^41^ The substantial economic burden of cancer survivorship may result in less attention on the oral health and dental care. Additionally, the shortage of dental medical resources may also contribute to the insufficient dental care, because the ratio of dentist‐to‐population was 1:10 000 in China, far lower than Australia (15:10 000) and US (16:10 000).^41^ High cost of oral health care and difficulty in regular dental visit remains in China, and even be more serious problems for Chinese cancer survivors.

Several public health strategies should be adopted to improve oral health among cancer survivors. Increase the number of dentists and oral health institution would be an important strategy to strengthen the Chinese oral health system. Cooperation of oncologist and dentist is needed to provide specific dental care for cancer survivors during their treatment and rehabilitation. Oral health education should be provided to promote cancer survivors’ oral health knowledge and culture proper oral hygiene habits.

Oral health is an important part of individual’ overall health, and it may contribute to individual’ QOL in both physically and psychologically. There are several domain‐specific scales that assess the oral health‐related quality of life (OHRQOL), such as General Oral Health Assessment Index (GOHAI)^43^ and Oral Health Impact Profile (OHIP).^44^ Most of these OHRQOL only reflect a part dimension of the overall QOL. For example, GOHAI focus on the physical changes that result from oral disease, and OHIP focus on the psychology and behavior dimension. Generic health‐related quality of life scale covers more dimensions than specific QOL scale and is more applicable to reflect the overall QOL. So we considered EORTC QLQ‐C30, a multiple cancer‐special scale, is applicable to reflect the overall QOL for cancer survivors. As far as we know, this is the first study to investigate the impact of toothbrushing and tooth loss on QOL among cancer survivors in large sample size. Some limitations of this study should also be acknowledged. First, some important information on clinical indicators such as cancer stage, metastatic and recurrence were not collected, which could potentially confound the impact of QOL. Second, the number of tooth loss was collected by self‐reported questionnaire, instead of oral health examination by experienced dentist. Because it is expensive to carry out oral health examination by experienced dentist in such a large‐scale epidemiologic study. So there was measurement error in our research. However, a previous research reported a high correlation between self‐reports and actual tooth number in the elderly,^45^ and we considered that the self‐reported tooth loss was valid, and use of self‐reported tooth loss could provide an accurate, easily obtained and economical measure of oral health. Last, we did not collect the information about the position of the missing teeth, the use of dentures, and the condition of the remaining teeth (dental caries, periodontal disease), which were also important indicators of oral health. Dental caries and periodontal disease are important factors that might result in poor QOL. The missing data of the remaining teeth (dental caries, periodontal disease) may result in overestimate the effect tooth loss on QOL. Wearing denture can act the function of chewing and improve self‐image. Denture may modify the negative effect of tooth loss on QOL. Without the status of denture, we may underestimate the actual effect of tooth loss on QOL. Future study needs to be done in order to carry out a more on these questions.

## 5 CONCLUSION

In conclusion, cancer survivors with frequent toothbrushing and less tooth loss experienced better QOL. Oral health care should also be brought to attention and be integrated into cancer care. Increased financial support on oral health care and effective methods to maintain the oral health might be helpful to improve the QOL for cancer survivors.

## CONFLICT OF INTEREST

The authors declare no potential conflict of interest.

## ETHICAL APRROVAL

The study was approved by the Medical Research Ethics Committee of the school of public health, Fudan University (The international registry NO. IRB00002408 & FWA00002399).
